# Substance Use Disorder Program Availability in Safety-Net and Non–Safety-Net Hospitals in the US

**DOI:** 10.1001/jamanetworkopen.2023.31243

**Published:** 2023-08-28

**Authors:** Ji E. Chang, Berkeley Franz, José A. Pagán, Zoe Lindenfeld, Cory E. Cronin

**Affiliations:** 1 Department of Public Health Policy and Management, School of Global Public Health, New York University, New York, New York; 2 Heritage College of Osteopathic Medicine, Ohio University, Athens; 3 College of Health Sciences and Professions, Ohio University, Athens

## Abstract

**Importance:**

Safety-net hospitals (SNHs) are ideal sites to deliver addiction treatment to patients with substance use disorders (SUDs), but the availability of these services within SNHs nationwide remains unknown.

**Objective:**

To examine differences in the delivery of different SUD programs in SNHs vs non-SNHs across the US and to determine whether these differences are increased in certain types of SNHs depending on ownership.

**Design, Setting, and Participants:**

This cross-sectional analysis used data from the 2021 American Hospital Association Annual Survey of Hospitals to examine the associations of safety-net status and ownership with the availability of SUD services at acute care hospitals in the US. Data analysis was performed from January to March 2022.

**Main Outcomes and Measures:**

This study used 2 survey questions from the American Hospital Association survey to determine the delivery of 5 hospital-based SUD services: screening, consultation, inpatient treatment services, outpatient treatment services, and medications for opioid use disorder (MOUD).

**Results:**

A total of 2846 hospitals were included: 409 were SNHs and 2437 were non-SNHs. The lowest proportion of hospitals reported offering inpatient treatment services (791 hospitals [27%]), followed by MOUD (1055 hospitals [37%]), and outpatient treatment services (1087 hospitals [38%]). The majority of hospitals reported offering consultation (1704 hospitals [60%]) and screening (2240 hospitals [79%]). In multivariable models, SNHs were significantly less likely to offer SUD services across all 5 categories of services (screening odds ratio [OR], 0.62 [95% CI, 0.48-0.76]; consultation OR, 0.62 [95% CI, 0.47-0.83]; inpatient services OR, 0.73 [95% CI, 0.55-0.97]; outpatient services OR, 0.76 [95% CI, 0.59-0.99]; MOUD OR, 0.6 [95% CI, 0.46-0.78]). With the exception of MOUD, public or for-profit SNHs did not differ significantly from their non-SNH counterparts. However, nonprofit SNHs were significantly less likely to offer all 5 SUD services compared with their non-SNH counterparts (screening OR, 0.52 [95% CI, 0.41-0.66]; consultation OR, 0.56 [95% CI, 0.44-0.73]; inpatient services OR, 0.45 [95% CI, 0.33-0.61]; outpatient services OR, 0.58 [95% CI, 0.44-0.76]; MOUD OR, 0.61 [95% CI, 0.46-0.79]).

**Conclusions and Relevance:**

In this cross-sectional study of SNHs and non-SNHs, SNHs had significantly lower odds of offering the full range of SUD services. These findings add to a growing body of research suggesting that SNHs may face additional barriers to offering SUD programs. Further research is needed to understand these barriers and to identify strategies that support the adoption of evidence-based SUD programs in SNH settings.

## Key Points

**Question:**

Are there differences in how safety-net vs non–safety-net hospitals report the availability of substance use disorder (SUD) services across the US?

**Findings:**

This cross-sectional analysis of 2846 hospitals from the 2021 American Hospital Association annual survey data found that safety-net hospitals have significantly lower odds of offering 5 hospital-based SUD services compared with non–safety-net hospitals.

**Meaning:**

These findings suggest that safety-net hospitals may face additional barriers to offering SUD programs compared with their non–safety-net counterparts, and further research is needed to understand these barriers and to identify strategies that support the adoption of evidence-based SUD programs in safety-net hospital settings.

## Teaser

This cross-sectional study examines differences in the delivery of different substance use disorder programs in safety-net hospitals vs non–safety-net hospitals across the US and evaluates whether these differences are increased in certain types of safety-net hospitals depending on ownership.

## Introduction

More than 106 000 people in the US died from a drug overdose in 2021, largely as a result of synthetic opioid use during the COVID-19 pandemic.^1^ This figure indicates a continued burden of substance use but also masks a disproportionate impact on underserved communities, where rates of overdose are increasing rapidly.^2,3^ The harms of substance use disorders (SUDs), including opioid use disorder (OUD), extend far beyond overdose, leading to stress in the health care system amid worsening health disparities.^3,4,5^

Hospitals have a crucial role to play in addressing the SUD crisis. Because patients with a history of injection drug use have high rates of secondary related infections (eg, endocarditis, hepatitis C virus, HIV, and skin and soft-tissue infections), hospitalization is a crucial juncture to engage patients in care. Hospitals support a range of services that includes formal treatment programs, such as partial hospitalization and withdrawal management in the inpatient setting and formal outpatient rehabilitation services. The evidence is strongest, however, for medication for OUD (MOUD) treatment with either buprenorphine or methadone.^6^ Given the important window of hospitalization, programs to identify SUD in hospitalized patients, initiate MOUD, and use warm handoffs to connect patients with treatment in the community hold particular promise. These models are often referred to as inpatient addiction consultation or liaison services, although a variety of hospital-supported models have been characterized in the literature.^7^ The addiction consultation model has demonstrated strong potential to improve morbidity and reduce mortality among patients with SUDs, although implementation challenges remain.^8,9,10,11,12^

These services may be even more critical in safety-net hospitals (SNHs), which disproportionately serve historically minoritized communities, Medicaid beneficiaries, the uninsured, and the underinsured.^13,14^ In underserved communities, hospitals are vital institutions tasked with providing preventive services and health care to communities.^9,15^ SUD-related admissions are more common in SNHs and are increasing rapidly.^11^ Given the location of SNHs in communities where substantial disparities exist, these hospitals are ideal sites to support the local opioid service infrastructure through their formal medical services and partnerships with outpatient organizations.

Prior studies^16,17^ of hospital-based SUD programs point toward multiple barriers to successful implementation, including financial strain, inadequate personnel, stigma, and perceived lack of expertise. Financial barriers are further complicated by factors such as limited community partnerships and stigma, which may also limit support for SUD programs.^18^ These barriers may be more pronounced in SNHs, which often have small operating margins and may face limitations on available resources to staff initiatives or to financially invest in sustaining successful efforts.^19,20^ For example, a qualitative study^12^ of an SNH emergency department (ED) identified multiple barriers to providing care for socially disadvantaged patients with SUDs, including the need to balance the needs of substance-involved patients with the necessity of managing limited resources. Successful adoption of SUD programs within SNH settings often entails obtaining seed funding from external partners and making a business case to executive leadership.^10^

SNHs may face unique barriers in offering SUD programs, and these difficulties have important implications for health equity. However, most studies to date have been qualitative or examined utilization within a single SNH setting, thus limiting generalizability.^11^ The aim of our study is 2-fold. First, our primary objective is to examine differences in the delivery of different SUD programs in SNHs vs non-SNHs across the US. Second, we conducted subpopulation analysis to investigate whether these differences are more pronounced in certain types of SNHs depending on their ownership. Although the vast majority of US hospitals are nonprofit, a sizable minority are publicly owned or for-profit. Previous research has found that ownership may impact hospital decisions to offer new services and technologies,^21,22,23^ hiring practices,^24^ hospital efficiency,^25,26^ and quality of care.^19,27^ Understanding these differences can help develop targeted interventions to support the implementation of a wider range of SUD services in hospitals serving safety-net populations.

## Methods

### Data Sources

In this cross-sectional study, we used the 2021 American Hospital Association (AHA) Annual Survey of Hospitals^28^ linked to County Health Rankings^29^ and Opioid Prescribing Data^30^ to examine the associations of safety net status and ownership with the availability of SUD services across hospitals. The AHA survey is an annual census of more than 6000 US hospitals and health care systems, with a response rate greater than 75% each year, and is a widely used source of data on US hospitals.^31^ Hospitals are identified by state, local, and national organizations, Medicare and Medicaid centers, and governmental bodies.^27^ This study was exempt from institutional review board approval and informed consent, because no human participant data were used, in accordance with 45 CFR §46, and it follows the Strengthening the Reporting of Observational Studies in Epidemiology (STROBE) reporting guidelines for cross-sectional studies.

### Dependent Variables

We used 2 questions from the AHA survey to determine the delivery of 5 hospital-based SUD services: screening, addiction consultation services, inpatient SUD services, outpatient SUD services, and MOUD.^29^ Screening and consultation were measured as a series of binary variables of service availability across 4 settings (ED, inpatient, primary care, or extended care) (eTable 1 in Supplement 1). Screening included but was not limited to the CAGE (Cut Down, Annoyed, Guilty, and Eye-Opener) Substance Abuse Screening Tool, the National Institute on Drug Abuse’s drug screening tool, and/or the TAPS (Tobacco, Alcohol, Prescription Medication, and Other Substance Use) Tool.^28^ Addiction consultation services were not further defined but refer to coordinated efforts within hospitals to reduce morbidity and mortality related to substance use through initiation of evidence-based treatment and successful transition of patients to community-based settings. We created separate binary outcome variables to indicate whether a hospital offered screening in any setting or consultation services in any setting.

MOUD, inpatient SUD services, and outpatient SUD services were measured as a series of questions on whether the health care organization offered services in any of their entities (hospital, health system, or joint venture). MOUD was defined as the use of use of medications approved by the Food and Drug Administration for the treatment of SUDs. Outpatient services were described as organized hospital services that provide medical care or services to outpatients for whom the primary diagnosis is alcoholism or other chemical dependency. Inpatient services were defined as organized hospital services providing intensive day or evening outpatient services of 3 hours’ duration or longer, distinguished from other outpatient visits of 1 hour. We created separate binary outcome variables to indicate whether a hospital offered MOUD, outpatient services, or inpatient services across any of the entities.

### Safety-Net Status and Ownership

Hospitals were defined as safety-net organizations if their percentage of Medicaid inpatient days was 1 SD above the state mean.^32^ We operationalized ownership as a categorical variable (1 = not-for-profit; 2 = public [nonfederal]; and 3 = for-profit).

### Organizational Variables

We measured hospital size as a categorical variable of the number of staffed hospital beds (<50, 50-199, 200-399, and ≥400 beds). We assessed teaching status with a binary variable (0 = independent; and 1 = part of the council of teaching hospitals). We measured religious affiliation with a binary variable.

### County Variables

We used the 2013 Rural-Urban Continuum Codes^33^ to classify counties as rural (codes 7-9) and nonrural (codes 1-6). We obtained the percentage of the population of White race in the county and the percentage of population who were unemployed using the 2019 American Community Survey. We obtained county-level overdose rates from the Centers for Disease Control and Prevention^34^ and opioid prescription rates (total number of opioid prescriptions per 100 persons) from amFAR.^35^

### Statistical Analysis

Data analysis was performed from January to March 2022. In descriptive analyses, we calculated the mean value of organizational and county variables, including the availability of SUD services, by SNH and non-SNH groups, and compared these values using *t* tests or χ^2^ tests. Next, we estimated 5 separate multilevel logistic regression models that accounted for the clustering of hospitals in states to examine differences in the availability of SUD services by safety-net status after controlling for organizational and environmental covariates. In secondary analysis, we ran separate regression models for screening and consultation, stratified by treatment setting, and MOUD, inpatient, and outpatient services stratified by treatment entity. Finally, we conducted subgroup analyses by estimating similar multilevel logistic regression models by the 3 ownership types. A 2-sided *P* < .05 calculated by the χ^2^ tests, *t* tests, and multivariable regression was considered statistically significant. Analyses were computed with Stata SE statistical software version 17 (StataCorp).^36^

## Results

After we excluded 1569 non–general medical hospitals, 209 federal hospitals, 55 hospitals located in territories, 1715 hospitals that did not respond to SUD treatment questions on the survey, and 37 hospitals with missing data on county variables, our analytic sample included 2846 hospitals. Comparisons of hospitals with and without missing data found that hospitals that did not answer the SUD survey questions were smaller (122 beds vs 190 beds) and were less likely to be teaching hospitals (2% vs 7%) and SNHs (5% vs 14%). A total of 409 hospitals (14.3%) were identified as SNHs, and 2437 (85.7%) were non-SNHs. Consistent with prior literature, the counties where SNHs were located had significantly higher overdose rates and unemployment, lower rates of White populations and opioid prescription rates, and were more likely to be classified as rural compared with counties without SNHs. SNHs also differed from non-SNHs in terms of organizational characteristics. Compared with non-SNHs, SNHs were larger (higher mean bed size), were more likely to be teaching hospitals, were more likely to be publicly owned, and were less likely to be a nonprofit or religious hospital (Table 1).

**Table 1.** Descriptive Statistics and Bivariate Comparison of SNHs and Non-SNHs

Characteristic	Hospitals, No. (%)			*P* value
	Total (N = 2846)	SNHs (n = 409)	Non-SNHs (n = 2437)	
Availability of SUD services				
Screening	2240 (79)	312 (76)	1928 (79)	.19
Consultation	1704 (60)	237 (58)	1467 (60)	.39
Inpatient SUD services	791 (27)	104 (25)	650 (27)	.59
Outpatient SUD services	1087 (38)	164 (40)	923 (38)	.39
Medications to treat opioid use disorder	1055 (37)	142 (35)	913 (37)	.28
Organizational characteristics				
Bed size, mean (SD)	192 (237)	244 (244)	183 (235)	<.001
Teaching hospital	207 (7)	63 (15)	144 (6)	<.001
Ownership				
Nonprofit	2010 (71)	261 (64)	1749 (72)	.001
Public	570 (20)	118 (29)	452 (19)	<.001
For-profit	266 (9)	30 (7)	236 (10)	.13
Religious hospital	339 (12)	26 (6)	313 (13)	<.001
County characteristics				
White population, mean (SD) %	77 (17)	74 (19)	78 (16)	<.001
Overdose rate, mean (SD) %	26 (16)	28 (20)	25 (16)	.01
Unemployment rate, mean (SD) %	7 (2)	8 (3)	7 (2)	.02
Rural classification	446 (16)	89 (22)	357 (15)	<.001
Opioid prescription rate, mean (SD) %	65 (30)	59 (31)	66 (31)	<.001

Abbreviations: SNH, safety-net hospital; SUD, substance use disorder.

Hospital participation in OUD programs ranged from 27% to 79%. The lowest proportion of hospitals reported offering inpatient SUD services (791 hospitals [27%]), followed by MOUD (1055 hospitals [37%]), and outpatient SUD services (1087 hospitals [38%]). The majority of hospitals reported offering consultation (1704 hospitals [60%]) and screening (2240 hospitals [79%]). In bivariate analyses, no differences emerged when comparing the availability of SUD services between SNHs and non-SNHs.

Multilevel logistic regression results indicate that, after controlling for organizational and environmental covariates, SNHs had significantly lower odds of offering all SUD services (screening odds ratio [OR], 0.62 [95% CI, 0.48-0.76]; consultation OR, 0.62 [95% CI, 0.47-0.83]; inpatient services OR, 0.73 [95% CI, 0.55-0.97]; outpatient services OR, 0.76 [95% CI, 0.59-0.99]; MOUD OR, 0.60 [95% CI, 0.46-0.78]) (Table 2). In secondary analysis stratified by setting, we found that with the exception of screening in extended care, SNHs had lower odds of offering screening and consultation across all settings, although statistical significance remained only for screening in the ED, consultation in the ED, and inpatient consultation (eTable 2 in Supplement 1). In general, overdose rates were positively associated with SUD services, whereas opioid prescription rates were negatively associated with SUD services (eTable 3 in Supplement 1). The county’s racial makeup (percentage of the population of White race) was not associated with screening or consultation, but was negatively associated with inpatient, outpatient, and MOUD services. Several organizational covariates, including bed size and ownership, were also statistically significant, such that in general, larger hospitals (more beds) had higher odds of offering SUD services, whereas public and for-profit hospitals had lower odds of offering SUD services compared with nonprofit hospitals.

**Table 2.** Adjusted Regression Results for Substance Use Disorder Service Provision by Safety-Net Status^a^

Variable	OR (95% CI)				
	Screening	Consultation	Inpatient	Outpatient	MOUD
Safety-net hospital	0.62 (0.48-0.76)^b^	0.62 (0.47-0.83)^b^	0.73 (0.55-0.97)^c^	0.76 (0.59-0.99)^c^	0.60 (0.46-0.78)^b^
Organizational characteristics					
Bed size					
<50	1 [Reference]	1 [Reference]	1 [Reference]	1 [Reference]	1 [Reference]
50-199	1.73 (1.39-2.16)^b^	1.35 (1.06-1.72)^c^	2.16 (1.63-2.86)^b^	1.95 (1.52-2.50)^b^	1.55 (1.22-1.98)^b^
200-399	2.67 (2.01-3.54)^b^	1.77 (1.28-2.46)^b^	2.96 (2.16-4.07)^b^	3.40 (2.53-4.58)^b^	2.37 (1.78-3.17)^b^
≥400	3.85 (2.63-5.62)^b^	2.34 (1.50-3.64)^b^	3.93 (2.71-5.71)^b^	6.38 (4.41-9.25)^b^	3.25 (2.28-4.65)^b^
Teaching hospital	1.51 (0.92-2.47)	1.14 (0.65-1.99)	0.75 (0.51-1.10)	1.25 (0.82-1.90)	1.92 (1.27-2.91)^b^
Ownership					
Nonprofit	1 [Reference]	1 [Reference]	1 [Reference]	1 [Reference]	1 [Reference]
Public	0.52 (0.41-0.66)^b^	0.56 (0.44-0.73)^b^	0.45 (0.33-0.61)^b^	0.58 (0.44-0.76)^b^	0.61 (0.46-0.79)^b^
For-profit	0.33 (0.24-0.46)^b^	0.40 (0.29-0.55)^b^	0.24 (0.15-0.38)^b^	0.15 (0.094-0.24)^b^	0.18 (0.12-0.29)^b^
Religious hospital	0.82 (0.62-1.08)	0.86 (0.62-1.19)	0.94 (0.70-1.25)	0.81 (0.61-1.07)	0.64 (0.48-0.85)^b^
County characteristics					
Percentage White population	1.00 (0.99-1.00)	1.00 (0.99-1.01)	0.98 (0.98-0.99)^b^	0.99 (0.98-0.99)^c^	0.99 (0.98-0.99)^c^
Overdose rate	3.90 (1.73-8.78)^b^	2.17 (0.89-5.26)^d^	2.77 (1.32-5.81)^b^	2.67 (1.25-5.67)^c^	4.50 (2.11-9.64)^b^
Percentage unemployed population	0.96 (0.91-1.02)	1.06 (1.00-1.13)^d^	0.95 (0.89-1.02)	0.98 (0.92-1.04)	0.97 (0.91-1.04)
Rural classification	0.81 (0.63-1.05)	0.96 (0.712-1.27)	0.49 (0.33-0.73)^b^	0.57 (0.41-0.78)^b^	0.57 (0.41-0.78)^b^
Opioid prescription rate	0.60 (0.43-0.85)^b^	0.89 (0.62-1.26)	0.45 (0.29-0.71)^b^	0.42 (0.28-0.64)^b^	0.47 (0.31-0.70)^b^

Abbreviations: MOUD, medications to treat opioid use disorder; OR, odds ratio.

^a^ Data are shown for 2846 observations and 50 groups.

^b^ *P* < .001.

^c^ *P* < .01.

^d^ *P* < .05.

To further examine these findings, we conducted subgroup analyses by ownership type by estimating multilevel logistic regression models by ownership categories (eTable 4 in Supplement 1). The majority of hospitals (2010 hospitals [71%]) were nonprofit hospitals, whereas one-fifth (570 hospitals [20%]) were public hospitals. Only 266 hospitals (9%) were for-profit hospitals. Subgroup analyses revealed a marked difference in the association of safety net status with the delivery of SUD services by ownership type. With the exception of MOUD, public or for-profit SNHs did not differ significantly from their non-SNH counterparts. However, nonprofit SNHs were significantly less likely to offer all 5 SUD services compared with their non-SNH counterparts (screening OR, 0.52 [95% CI, 0.41-0.66]; consultation OR, 0.56 [95% CI, 0.44-0.73]; inpatient services OR, 0.45 [95% CI, 0.33-0.61]; outpatient services OR, 0.58 [95% CI, 0.44-0.76]; MOUD OR, 0.61 [95% CI, 0.46-0.79]) (Figure).

**Figure.**
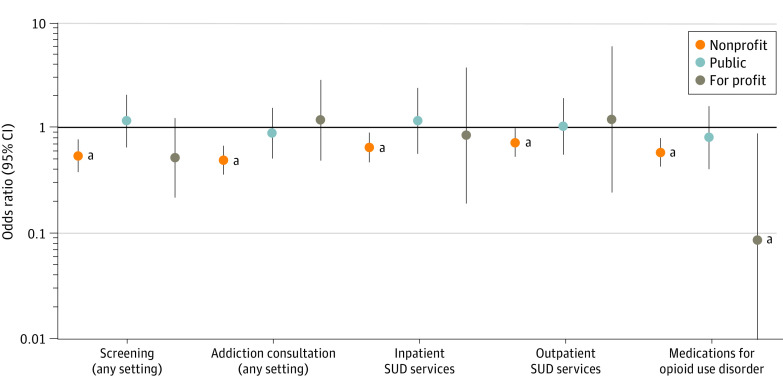
Comparison of Substance Use Disorder (SUD) Services Between Safety-Net and Non–Safety-Net Hospitals by Ownership Type (Nonprofit, Public, and For-Profit) Graph depicts the odds of offering hospital-based SUD services for safety-net hospitals (reference category, non–safety net hospitals) by ownership type. Definitions of screening, addiction consultation, inpatient SUD services, outpatient SUD services, and medications for opioid use disorder appear in the Methods section. ^a^*P* < .05.

## Discussion

In this cross-sectional study of US hospitals, we found marked variation in the types of SUD services offered, with only approximately one-third offering MOUD, suggesting a critical underutilization of evidence-based SUD programs by hospitals. These findings are consistent with prior studies^37,38,39^ pointing to limited availability and utilization of MOUD in hospitals. For instance, an audit study^37^ of all acute care hospitals in New Mexico found that nearly one-half (45.5%) of surveyed hospitals did not have buprenorphine or naloxone on their inpatient formularies. Studies^38^ of MOUD prescribing in Veterans Affairs hospitals found that only 15% of patients with OUD received opioid agonist therapy, whereas a study^39^ of 143 hospitals across 21 states found that MOUD was initiated among only 19% of adults with OUD during hospitalization. These findings highlight the importance of addressing barriers and expanding access to evidence-based SUD programs in hospitals.

Although the raw figures showed comparable rates of SUD program adoption between SNHs and non-SNHs, adjusted models revealed stark differences. Across the board, SNHs have significantly lower odds of offering all 5 SUD services after controlling for other organizational and environmental factors. These results add to a growing body of research suggesting that SNHs may face additional barriers to offering SUD programs.^12,40^ Moreover, we observed significant differences based on hospital ownership type. Public and for-profit hospitals, compared with nonprofit hospitals, had lower odds of offering all 5 OUD services. For example, the odds of offering MOUD were 5 times higher in nonprofit hospitals than in for-profit hospitals. Although the underlying reasons for this disparity require further investigation, possible factors could include variations in organizational priorities and access to specialized staff and resources. These findings highlight the need to address barriers and disparities in the provision of SUD services across different hospital ownership types to ensure equitable access to evidence-based care for individuals with OUD.

Results from our subgroup analysis further illuminate differences in program adoption by safety-net status and ownership type. Although safety-net status was not significantly associated with a difference in programming between for-profit and public hospitals, among nonprofit hospitals, SNHs were significantly less likely to offer all 5 SUD services. These differences suggest interacting factors between ownership and safety-net status that limit SUD service implementation. In other words, safety-net status matters, but appears to be a more prominent factor for nonprofit hospitals. These findings are concerning given that, compared with non-SNHs, nonprofit SNHs are more likely to lose funding from safety-net subsidy programs,^41^ be penalized for patient readmissions,^42^ and have a higher uncompensated care burden.^43^ This may limit the ability of nonprofit SNHs to offer additional services, as well as the funds available for hiring needed personnel.

The factors underlying the findings of our study are multifaceted, involving challenges at the clinician, hospital, and policy levels.^44^ From a policy perspective, it is crucial to address the financial challenges faced by nonprofit SNHs to ensure equitable access to SUD services. This may involve targeted funding initiatives, reimbursement models that adequately account for the unique circumstances of SNHs, and policy efforts to reduce the uncompensated care burden. In addition, strategies should be developed to support workforce development and recruitment of personnel with expertise in addiction medicine within nonprofit SNHs. This support can include technical assistance, training, and resources to enhance the capacity of hospitals and clinicians in delivering evidence-based care and addressing challenges in implementing SUD services.

### Limitations

There are several limitations to our study. First, there is wide variation in the conceptualization and definition of SNHs in the literature.^9,27^ Although our approach is consistent with a commonly adopted operationalization of safety-net status, using an alternative definition may lead to different results. Second, because the AHA Annual Survey relies on self-reported data by hospital administrators, there might be biases in the responses, such as overreporting or underreporting of certain services. As a result, there may be nonrandom differences in how different types of hospitals respond to the survey. For example, SNHs, which predominantly serve socioeconomically and medically vulnerable populations, might have a better understanding of patient needs and, thus, might respond differently regarding the services they offer. This potential bias in responses from SNHs could affect the findings and comparisons involving these hospitals. Similarly, the AHA survey data primarily indicate the availability of services in a binary form (yes or no), without providing information on the scale, scope, or adequacy of these services. Consequently, there is a possibility that the reported availability of services does not accurately represent the depth or extent of care provided. For example, a hospital with a single addiction specialist available on a limited basis may report the availability of addiction treatment services, even though it may not meet the needs of a large patient population. These binary responses mask the nuances in service provision that reflect differences in the actual delivery of SUD care.

Moreover, the lack of granularity in the data restricts the ability to assess the quality and comprehensiveness of services offered, particularly in SNHs, where the demand for certain services may be higher. In addition, although we used available data in the AHA survey, it is possible that important explanatory variables (ie, financial information) were not included in our analysis. These omitted variables could introduce bias and affect the observed associations. Future research should strive to incorporate a broader range of relevant factors to better understand the complex determinants of SUD service provision and mitigate potential biases associated with omitted variables. Similarly, the exclusion of hospitals that did not respond to the AHA survey or the portion of the survey on SUD service provision may introduce a nonresponse bias. Furthermore, the observational, cross-sectional nature of our study limits our ability to determine causality. Replication of this study using longitudinal data could enhance confidence in our findings between safety-net status, ownership, and the availability of SUD programs.

It is worth noting that our data come from the 2021 AHA Annual Survey of Hospitals, the first full year of data collected following the onset of the COVID-19 pandemic. The COVID-19 pandemic put extreme stress on the health care system in the form of acute staffing shortages and ongoing financial strains. These challenges may have been amplified in safety-net systems, given their role in serving low-income communities that were most affected by the COVID-19 pandemic. Given the cross-sectional nature of our study, it is unclear whether our findings reflect a temporary strain on hospital resources during a public health emergency or entrenched differences in SUD program adoption. Further research is needed to assess patterns in service provisions over time.

## Conclusions

SNHs are ideal sites to deliver addiction treatment to patients with SUDs. Yet, SNHs had significantly lower odds of offering the full range of SUD services compared with their non-SNH counterparts. These findings underscore the urgent need to address the underresourcing and systemic challenges faced by SNHs, especially nonprofit institutions, to ensure equitable access to evidence-based SUD care. Efforts must be directed toward developing targeted interventions, policy reforms, and resource allocation strategies to bridge the gap in SUD services and promote equitable health care delivery.

## Notes

Supplement 1. **eTable 1.** Descriptive Statistics of Substance Use Disorder Screening and Consultation Stratified by Setting (n=2,846) **eTable 2.** Adjusted Regression Results for Substance Use Disorder Screening and Consultation Stratified by Setting **eTable 3.** Descriptive Statistics of Medications for Opioid Use Disorder (MOUD), Inpatient Services, and Outpatient Services by Entity (N=2,846) **eTable 4.** Adjusted Regression Results for Medications for Opioid Use Disorder (MOUD), Inpatient Services, and Outpatient Services by Entity

Supplement 2.
Data Sharing Statement
