# Supplementary material for: Substance Use Disorder Program Availability in Safety-Net and Non–Safety-Net Hospitals in the US
Source: JAMA Netw Open. 2023 Aug 28;6(8):e2331243. doi: 10.1001/jamanetworkopen.2023.31243 (PMC10463097; doi:10.1001/jamanetworkopen.2023.31243)
Supplement: Supplement 1. — eTable 1. Descriptive Statistics of Substance Use Disorder Screening and Consultation Stratified by Setting (n=2,846) eTable 2. Adjusted Regression Results for Substance Use Disorder Screening and Consultation Stratified by Setting eTable 3. Descriptive Statistics of Medications for Opioid Use Disorder (MOUD), Inpatient Services, and Outpatient Services by Entity (N=2,846) eTable 4. Adjusted Regression Results for Medications for Opioid Use Disorder (MOUD), Inpatient Services, and Outpatient Services by Entity [file jamanetwopen-e2331243-s001.pdf]

Supplemental Online Content

Chang JE, Franz B, Pagán JA, Lindenfeld Z, Cronin C. Substance use disorder program availability in safety-net and non–safety-net hospitals in the US. *JAMA Netw Open*. 2023;6(8):e2331243. doi:10.1001/jamanetworkopen.2023.31243

eTable 1. Descriptive Statistics of Substance Use Disorder Screening and Consultation Stratified by Setting (n=2,846)
eTable 2. Adjusted Regression Results for Substance Use Disorder Screening and Consultation Stratified by Setting
eTable 3. Descriptive Statistics of Medications for Opioid Use Disorder (MOUD), Inpatient Services, and Outpatient Services by Entity (N=2,846)
eTable 4. Adjusted Regression Results for Medications for Opioid Use Disorder (MOUD), Inpatient Services, and Outpatient Services by Entity

This supplemental material has been provided by the authors to give readers additional information about their work.

© 2023 Chang JE et al. *JAMA Network Open.*

eTable 1. Descriptive Statistics of Substance Use Disorder Screening and Consultation Stratified by Setting (n=2,846)

	**Screening (No. (%))**	**Consultation (No. (%))**
**Inpatient**	1,902 (66.8%)	1,328 (46.7%)
**Emergency Department**	2,104 (73.9%)	1,530 (53.8%)
**Primary Care**	1,680 (59.0%)	1,124 (39.5%)
**Extended Care**	607 (21.3%)	377 (13.6%)

© 2023 Chang JE et al. *JAMA Network Open.*

eTable 2. Adjusted Regression Results for Substance Use Disorder Screening and Consultation Stratified by Setting

	**Screening** (OR (95% CI))				**Consultation** (OR (95% CI))			
	**Inpatient**	**ED**	**Primary Care**	**Extended Care**	**Inpatient**	**ED**	**Primary Care**	**Extended Care**
Safety Net Hospital	0.84 (0.65 – 1.08)	0.71** (0.54 - 0.92)	0.82 (0.64 – 1.04)	1.33* (1.02 – 1.73)	0.67** (0.52 – 0.86)	0.64*** (0.50 – 0.83)	0.84 (0.66 – 1.07)	0.98 (0.71 – 1.35)
Organizational Characteristics								
Bed Size (Ref: <50)								
50-199	1.82*** (1.46- 2.25)	1.57*** (1.25 - 1.98)	1.15 (0.93 – 1.43)	1.22 (0.95 – 1.56)	2.66*** (2,12 – 3.35)	1.92*** (1.54 – 2.39)	1.30* (1.04 – 1.63)	1.87*** (1.33 – 2.62)
200-399	2.41*** (1.82 - 3.19)	2.16*** (1.59 – 2.92)	1.12 (0.86 – 1.45)	1.05 (0.76 – 1.45)	4.60*** (3.45 – 6.11)	3.10*** (2.35 – 4.10)	1.81*** (1.38 – 2.38)	2.26*** (1.53 – 3.34)
400+	3.06*** (2.11 - 4.43)	2.23*** (1.52 – 3.31)	1.96*** (1.39 – 2.76)	2.11*** (1.45 – 3.07)	6.78*** (4.72 – 9.74)	4.00*** (2.79 – 5.72)	2.50*** (1.78 – 3.51)	4.17*** (2.69 – 6.47)
Teaching Hospital	1.20 (0.76 – 1.91)	1.12 (0.70 – 1.82)	1.50 (0.99 – 2.27)	0.78 (0.51 – 1.19)	1.72* (1.10 – 2.70)	0.88 (0.90 – 2.12)	1.62* (1.11 – 2.38)	0.82 (0.53 – 1.26)
Ownership (Ref: Nonprofit)								
Public	0.65*** (0.51 - 0.81)	0.69** (0.54 - 0.88)	0.85 (0.68 – 1.06)	0.98 (0.75- 1.28)	0.51*** (0.40 - 0.65)	0.62*** (0.50 – 0.79)	0.80 (0.63 – 1.01)	0.79 (0.56 – 1.10)
For-profit	0.49*** (0.37 - 0.66)	0.52*** (0.38- 0.71)	0.32*** (0.23 – 0.43)	0.56** (0.37 - 0.86)	0.36*** (0.26 - 0.51)	0.40*** (0.29 – 0.54)	0.23*** (0.15 – 0.34)	0.29*** (0.15 – 0.53)
Religious Hospital	0.99 (0.75 - 1.31)	1.02 (0.75 - 1.34)	0.82 (0.63 – 1.06)	0.91 (0.67 - 1.24)	0.69** (0.52 - 0.90)	0.88 (0.67 – 1.15)	0.87 (0.67 – 1.13)	0.54** (0.35 – 0.81)
County Characteristics								
Percent White	1.00 (0.74 - 1.31)	1.00 (0.99 - 1.00)	1.00 (0.99 – 1.01)	1.01* (1.00- 1.02)	0.99 (0.99 – 1.00)	0.99 (0.99 – 1.00)	1.00 (0.99 – 1.00)	1.02 (1.00 – 1.02)
Overdose rate	1.84 (0.89- 3.79)	1.57 (0.70 – 3.50)	1.56 (0.82 – 3.01)	0.76 (0.35 – 1.68)	3.95*** (1.85 – 8.46)	2.56* (1.18 – 5.58)	1.00 (0.99 – 1.00)	1.38 (0.62 – 3.09)

© 2023 Chang JE et al. *JAMA Network Open.*

Percent unemployed	1.03 (0.98 - 1.01)	1.06* (1.00 - 1.13)	1.01 (0.96 – 1.07)	1.02 (0.96 - 1.08)	0.94* (0.87 – 1.00)	0.98 (0.93 – 1.04)	0.97 (0.91 – 1.03)	1.05 (0.97 – 1.12)
Rural classification	0.92 (0.71 - 1.19)	0.99 (0.75 - 1.29)	1.19 (0.92 – 1.54)	1.49** (1.12 – 1.97)	0.75 (0.56– 1.00)	0.77 (0.59 – 1.00)	0.97 (0.74 – 1.27)	0.78 (0.51 – 1.20)
Opioid prescription rate	0.96 (0.70 – 1.32)	0.84 (0.60 - 1.17)	0.88 (0.64 – 1.21)	0.93 (0.64 – 1.34)	0.55** (0.38 - 0.80)	0.59** (0.42 – 0.84)	0.62* (0.43 – 0.90)	0.57* (0.35 – 0.93)
Observations	2,846	2,846	2,846	2,846	2,846	2,846	2,846	2,846
Groups	50	50	50	50	50	50	50	50

*p<0.05

**p<0.01

***p<0.001
ED=Emergency Department

© 2023 Chang JE et al. *JAMA Network Open.*

eTable 3. Descriptive Statistics of Medications for Opioid Use Disorder (MOUD), Inpatient Services, and Outpatient Services by Entity (N=2,846)

	MOUD (No. (%))	Inpatient Services (No. (%))	Outpatient Services (No. (%))
**Hospital**	515 (18.1%)	220 (7.7%)	470 (16.5%)
**Health System**	569 (20.0%)	488 (17.2%)	616 (21.6%)
**Joint Venture**	102 (3.6%)	97 (3.4%)	130 (4.6%)

MOUD = Medications for opioid use disorder

eTable 4: Adjusted Regression Results for Medications for Opioid Use Disorder (MOUD), Inpatient Services, and Outpatient Services by Entity

	MOUD			Inpatient Services			Outpatient Services		
	**Hospital**	**Health System**	**Joint Venture**	**Hospital**	**Health System**	**Joint Venture**	**Hospital**	**Health System**	**Joint Venture**
Safety Net Hospital	0.87 (0.64 – 1.18)	0.50*** (0.36 - 0.71)	0.70 (0.36 – 1.36)	1.16 (0.78 – 1.71)	0.50*** (0.35 – 0.72)	1.03 (0.56 – 1.92)	1.32 (0.98 – 1.79)	0.39*** (0.28 – 0.55)	0.96 (0.56 – 1.65)
Organizational Characteristics									
Bed Size (Ref: <50)									
50-199	1.67** (1.21-2.32)	1.44* (1.07 - 1.94)	1.01 (0.58 – 1.75)	2.63** (1.49 – 4.66)	2.06*** (1.47 – 2.89)	1.23 (0.68 – 2.20)	2.08*** (1.43 – 3.00)	1.67** (1.24 – 2.24)	1.17 (0.70 – 1.97)
200-399	2.77*** (1.92 – 4.00)	1.71** (1.22 – 2.40)	0.79 (0.40 – 1.58)	6.94*** (3.83 – 12.58)	2.27*** (1.56 – 3.29)	0.98 (0.48 – 1.99)	4.62*** (3.07 – 6.95)	1.94*** (1.39 – 2.71)	1.18 (0.63 – 2.22)
400+	5.16*** (3.40 – 7.82)	1.28 (0.85 – 1.93)	127 (0.58 – 2.74)	15.00*** (8.00 – 28.18)	1.73** (1.10 – 2.71)	1.39 (0.30 – 3.11)	12.97*** (8.24 – 20.42)	1.44 (0.96 – 2.16)	2.23* (1.11 – 4.46)
Teaching Hospital	2.89*** (1.56 – 3.36)	0.79 (0.52 – 1.21)	1.03 (0.43 – 1.42)	1.06 (0.67 – 1.68)	0.79 (0.51 – 1.22)	0.72 (0.30 – 1.73)	1.47 (0.99 – 2.19)	0.82 (0.54 – 1.25)	1.22 (0.62 – 2.40)
Ownership (Ref: Nonprofit)									
Public	1.10 (0.80 – 1.51)	0.25*** (0.17 - 0.38)	0.79 (0.43 – 1.42)	0.77 (0.48-1.23)	0.39*** (0.26 - 0.58)	0.46* (0.22 – 0.93)	1.11 (0.79 – 1.56)	0.32*** (0.22 – 0.47)	0.60 (0.33 – 1.08)
For-profit	0.18***	0.16***	0.66	0.78	0.13***	0.38	0.24***	0.15***	0.48 (0.19 – 1.25)

© 2023 Chang JE et al. *JAMA Network Open.*

	(0.08 - 0.40)	(0.09- 0.29)	(0.27 – 1.61)	(0.39 – 1.54)	(0.07 - 0.27)	(0.13 – 1.10)	(0.11 – 0.50)	(0.08 – 0.27)	
Religious Hospital	0.78 (0.54 - 1.10)	0.60** (0.43 – 0.84)	0.18** (0.06 – 0.59)	1.06 (0.68 - 1.65)	0.96 (0.70 – 1.33)	0.55 (0.26 – 1.15)	0.86 (0.60 – 1.24)	0.81 (0.60 – 1.10)	0.72 (0.38 – 1.08)
County Characteristics									
Percent White	1.01 (1.00 - 1.02)	0.98 (0.97 - 0.99)	1.00 (0.99 – 1.02)	1.01 (0.99- 1.02)	0.98*** (0.97 – 0.99)	0.99 (0.97 – 1.01)	1.00 (0.99 – 1.02)	0.98*** (0.97 – 0.99)	1.02** (1.01 – 1.04)
Overdose rate	4.41*** (2.05- 9.51)	2.20* (1.00 – 4.83)	1.12 (0.25 – 4.90)	3.38** (1.35 – 8.47)	2.39* (1.06 – 5.41)	1.26 (0.30 – 5.27)	2.85* (1.24 – 6.56)	2.63* (1.21 – 5.70)	2.91 (0.88 – 9.63)
Percent unemployed	1.01 (0.94 - 1.02)	0.97 (0.91 - 1.05)	1.06 (0.94 – 1.21)	0.95 (0.87 - 1.05)	0.95 (0.88 – 1.03)	1.01 (0.88 – 1.16)	0.96 (0.88 – 1.04)	0.98 (0.92 – 1.05)	1.13* (1.01 – 1.26)
Rural classification	0.73 (0.49 - 1.10)	0.47** (0.30 - 0.74)	0.71 (0.33 – 1.51)	0.87 (0.44 – 1.72)	0.36*** (0.21– 0.63)	0.76 (0.32 – 1.77)	0.66 (0.42 – 1.03)	0.48** (0.31 – 0.75)	1.20 (0.64 – 2.25)
Opioid prescription rate	0.61 (0.36 – 1.04)	0.28*** (0.17 - 0.47)	1.53 (0.68 – 3.46)	0.68 (0.35 – 1.32)	0.41** (0.24 - 0.71)	0.40 (0.15 – 1.10)	0.65 (0.37 – 1.16)	0.31*** (0.19 – 0.51)	0.37* (0.16 – 0.86)
Observations	2,846	2,846	2,846	2,846	2,846	2,846	2,846	2,846	2,846
Groups	50	50	50	50	50	50	50	50	50

*p<0.05

**p<0.01

***p<0.001

© 2023 Chang JE et al. *JAMA Network Open.*
