# Supplementary material for: Substance Use Disorder Program Availability in Safety-Net and Non–Safety-Net Hospitals in the US
Source: JAMA Netw Open. 2023 Aug 28;6(8):e2331243. doi: 10.1001/jamanetworkopen.2023.31243 (PMC10463097; doi:10.1001/jamanetworkopen.2023.31243)
Supplement: Supplement 2. — Data Sharing Statement [file jamanetwopen-e2331243-s002.pdf]

Data Sharing Statement

Chang. Substance Use Disorder Program Availability in Safety-Net and Non–Safety-Net Hospitals in the US. *JAMA Netw Open*. Published August 28, 2023.
doi:10.1001/jamanetworkopen.2023.31243

Data
Data available: No

Additional Information
Explanation for why data not available: Data must be purchased from the American Hospital Association to access and share.
